# Building Synthetic Sterols Computationally – Unlocking the Secrets of Evolution?

**DOI:** 10.3389/fbioe.2015.00121

**Published:** 2015-08-21

**Authors:** Tomasz Róg, Sanja Pöyry, Ilpo Vattulainen

**Affiliations:** ^1^Department of Physics, Tampere University of Technology, Tampere, Finland; ^2^MEMPHYS-Center for Biomembrane Physics, University of Southern Denmark, Odense, Denmark

**Keywords:** cholesterol, synthetic sterol, computer simulation, molecular dynamics simulation

## Abstract

Cholesterol is vital in regulating the physical properties of animal cell membranes. While it remains unclear what renders cholesterol so unique, it is known that other sterols are less capable in modulating membrane properties, and there are membrane proteins whose function is dependent on cholesterol. Practical applications of cholesterol include its use in liposomes in drug delivery and cosmetics, cholesterol-based detergents in membrane protein crystallography, its fluorescent analogs in studies of cholesterol transport in cells and tissues, etc. Clearly, in spite of their difficult synthesis, producing the synthetic analogs of cholesterol is of great commercial and scientific interest. In this article, we discuss how synthetic sterols non-existent in nature can be used to elucidate the roles of cholesterol’s structural elements. To this end, we discuss recent atomistic molecular dynamics simulation studies that have predicted new synthetic sterols with properties comparable to those of cholesterol. We also discuss more recent experimental studies that have vindicated these predictions. The paper highlights the strength of computational simulations in making predictions for synthetic biology, thereby guiding experiments.

## Why Synthetic Lipids and Sterols are Important?

As nature has designed thousands of lipid species, why then would we need synthetic lipids in addition? Clearly, however, the use of synthetic lipids is commonplace in both applied and basic sciences. The largest applications of synthetic lipids are in pharmacology, where synthetic lipids are used, e.g., in drug delivery and gene transfection. In drug delivery, the most commonly used carriers are liposomes, however, simple micelles or nanodiscs can be used as well. Technical requirements for the carriers include optimal lifetime, just-in-time triggered release of their contents, feasible targeting agents, etc. Numerous synthetic lipids have been synthesized and tested for this purpose [for a recent review, see Kohli et al. (2014)]. As in several other cases, here also atomistic molecular dynamics simulations have been used to unravel the physicochemical properties of these lipids [e.g., Bunker (2012)]. In gene transfection, one possible form of DNA packaging is the so-called *genosome*, commonly also called the *lipoplex*. Lipoplex is an aggregate of DNA and lipids; however, the cationic lipids needed to form this aggregate do not exist in nature. Consequently, only synthetic lipids can be used for this purpose.

Synthetic lipids have also numerous applications in basic research. Possibly, the most apparent example is labeling lipids with fluorescent or spin labels. For instance, cholesterol labeled with BODIPY or NBD has been used to study cholesterol trafficking in cells. Here also, molecular dynamics (MD) simulations have been used to examine the different behaviors of native and modified molecules, thus complementing and explaining experiments (Hölttävuori et al., 2008; Robalo et al., 2013). Synthetic detergents like cholesteryl hemisuccinate are commonly used in G-protein coupled receptor crystallography, and again MD simulations have elucidated the differences between native and modified molecules (Kulig et al., 2014, 2015). More sophisticated applications of synthetic lipids include modifying the molecule’s native structure by removing functional groups, in order to understand their individual function. Particularly, sphingolipids have been extensively studied in this manner (Slotte, 2013).

In this perspective article, we show an example of this last approach. The studies discussed in this article aimed at understanding the detailed structure–function relationships of cholesterol, in particular, the role of methyl groups attached to the steroid ring system. As we next explain in detail, these groups might with good reasons be thought of as unnecessary molecular fossils. However, as the below discussion highlights, extensive atomistic MD simulations showed that the methyl groups are indeed important parts of the cholesterol molecule, and the simulation results were later confirmed by experiments.

## What is so Special about Cholesterol?

Cholesterol is a truly special molecule and absolutely vital for animals’ wellbeing. This is probably best proved by the complete lack of mutations that would totally block the synthesis of cholesterol. Furthermore, some rare genetic syndromes caused by impaired cholesterol synthesis lead to serious conditions or death (Kelley and Herman, 2001). To ensure proper function, cholesterol needs a high degree of structural specificity. Indeed, cholesterol’s precursors that have one additional double bond compared to cholesterol cannot substitute it independently, irrespective of whether the bond is located in the ring structure (7-dehydrocholesterol) or in the hydrocarbon tail (desmosterol) (Kelley and Herman, 2001). Highlighting its pivotal role, cholesterol is the single most common lipid species in our body. Its concentration in cell membranes varies from 30 to 50 mol% (van Meer et al., 2008), whereas in specialized membranes, such as the ocular lens (Mason et al., 2003), its concentration may reach 75 mol%. Ten percent of brain dry mass is cholesterol (Snipes and Suter, 1997). In the intracellular membranes, the concentration of cholesterol is lower but still typically 10–20 mol%. Deservedly, cholesterol is one of the most studied lipid molecules of all time.

Many of the various functions of cholesterol are related to modifying the structural properties of membranes. For example, cholesterol increases the mechanical strength of membranes, decreases their permeability, and affects membrane thickness and condensation [for reviews, see Ohvo-Rekila et al. (2002), Almeida (2009), and Róg and Vattulainen (2014)]. Presence of cholesterol alters the pressure profile across membranes (Ollila et al., 2007); this effect is sensitive to even small modifications in sterol structure. Cholesterol also modulates the phase behavior of lipid bilayers in a complex way (Ipsen et al., 1987; Vist and Davis, 1990). At larger cholesterol concentrations, a new phase called the *liquid ordered (Lo) phase* occurs, while at lower concentrations a *liquid disordered phase* is observed. Cholesterol is able to promote the formation of so-called *lipid rafts*, functional nanoscale domains that are rich in cholesterol, sphingolipids, and saturated phospholipids (Lingwood and Simons, 2010), and numerous cellular functions, such as signaling and intracellular trafficking, actually depend on cholesterol (Coskun and Simons, 2011). Other cellular functions of cholesterol include its role as a metabolite and precursor of bile salts, some vitamins, and adrenal, pituitary, and sex (steroid) hormones.

All of the discussed points give rise to a picture of cholesterol having a very special and specific structure. Already during the seventies, cholesterol was established to be composed of three structural elements: a small hydroxyl head group, a rigid steroid ring system, and a short iso-octyl tail (Demel et al., 1972; Wenz, 2012). Modifications of these elements typically decrease the strength of cholesterol’s effects on the physical properties of lipid bilayers and, as mentioned above, other sterols cannot substitute cholesterol in its biological function.

## Does Cholesterol’s Biosynthetic Pathway Reflect Molecular Evolution?

**Figure 1:**
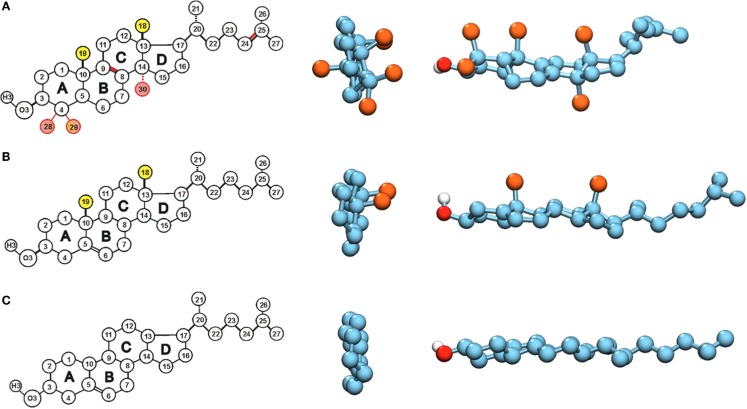
**Structures of (A) lanosterol, (B) cholesterol, and (C) Dchol**. Chemical structures and atom numberings are shown on the left. The sites where differences occur between lanosterol and cholesterol have been colored with pink, and the methyl groups that are removed in Dchol are marked in yellow. In the middle and on the right, space-filling models of the same molecules are given. The middle panel shows only the ring system of each sterol. The point of view is from the direction of the hydroxyl group, and the ring system lies on the perpendicular plane. The off-plane methyl groups are colored in orange, and carbons in cyan. On the right, space-filling models of the whole sterol molecules are shown as side views, the ring system lying on the horizontal plane. The off-plane methyl groups are colored in orange, carbons in cyan, oxygen in red, and hydrogen in silver.

The biosynthesis of cholesterol is a complex process. The first sterol on the path is lanosterol (Figure 1), which is synthesized from squalene in a reaction that requires molecular oxygen. Consequently, the occurrence of this sterol can be located in the history of earth to a time after prokaryotic life had developed. Thus, perhaps not surprisingly, sterols are not typical bacterial lipids with the exception of *Mycoplasma*, one of the most simple parasitic bacteria that utilizes lipids produced by their hosts and is actually often thought of as an intermediate form of life between viruses and bacteria. Next, lanosterol is converted into cholesterol through two alternative pathways: one ending in desmosterol and another with 7-dehydrocholesterol – the direct precursors of cholesterol. Although textbooks show these as separate pathways, it should be kept in mind that at each of the individual steps, it is possible to swap to the other pathway, as appropriate enzymes for this do exist. The conversion of lanosterol to cholesterol needs a minimum of only 7 steps; however, 18 steps are possible and thus also 18 enzymes exist! This has to be energetically very expensive for cells, once more stressing the great importance of cholesterol.

This amazing redundancy has been noticed a long time ago and it has given rise to a question as to what is so special about the structure of cholesterol that sets it apart from lanosterol and other precursors. When looking at the structures of lanosterol and cholesterol in Figure 1, one notices that the differences are limited to the number and position of double bonds (one more in lanosterol) and the number of methyl groups attached to the steroid ring system (three more in lanosterol). While these do not seem such large differences, they have substantial consequences. First, it has been shown that lanosterol does not induce the existence of the *Lo* phase and thus lipid rafts cannot be formed by this sterol (Miao et al., 2002). Even more intriguingly, it has been shown, already in the sixties, that cholesterol’s precursors affect the properties of lipid bilayers step by step more, ending in cholesterol whose effect is the strongest of all. Thus, it has been proposed that the biosynthetic pathway of cholesterol reflects the evolutionary optimization of its structure (Bloch, 1979; Nielsen et al., 2000).

This idea was the starting point for our first investigation into the matter using atomistic MD simulations. Intriguingly, the methyl groups stick out from one side of the cholesterol molecule, called the β-side, while the other side, called the α-side, is flat (Figure 1). Lanosterol has three additional methyl groups as compared to cholesterol. Two of these additional methyl groups stick out from the α-side, while the third is directed along the ring plane. Our first results showed greater ordering of saturated lipids neighboring the α-side of cholesterol as compared to lipids next to the β-side (Róg and Pasenkiewicz-Gierula, 2001). Subsequent studies showed that the packing of lipid carbon atoms near the α-side is tight; while near the β-side it is much looser (Róg and Pasenkiewicz-Gierula, 2004). In other words, we showed that the flatness of the ring is associated with higher ordering of lipids. These results fit perfectly with the idea of considering the removal of methyl groups as optimization of cholesterol’s structure.

At this point, another open question remains about the role of double bonds in the sterols’ structure. In case of desmosterol, atomistic MD studies showed it to be inferior to cholesterol in its ordering capability of saturated lipids; while in the case of unsaturated lipids, there is no significant difference between the two sterols (Vainio et al., 2006; Róg et al., 2008). These results agree with experimental data (Huster et al., 2005; Scheidt et al., 2005). Subsequently, studies of 7-dehydrocholesterol showed very small or non-existent differences as compared to cholesterol. This was observed both in MD simulations (Róg et al., 2008; Liu et al., 2011) and experimental studies (Chen and Tripp, 2012). However, there are two conjugated double bonds in the ring structure of 7-dehydrocholesterol, which may render the molecule prone to oxidation. This might be the reason why 7-dehydrocholesterol is not the sterol of choice for biological membranes.

Eukaryotic cells require the ordering properties of sterols. At the same time, all of the above considerations lead us to the conclusion that these ordering properties are decreased in the presence of methyl groups. Then, why would any methyl groups remain on the β-side of the ring system? Are they molecular fossils? Could we further optimize the structure of sterols by removing these last remaining groups?

## Are Cholesterol’s Methyl Groups Molecular Fossils? – Simulations Said No!

Molecular dynamics is a very flexible method and provides an inexpensive way to start investigating a new molecule. Surely, if the new molecule does not exist yet, validating the model may be problematic. Nevertheless, taking into account the current development of organic synthesis methods, one may expect the results from MD to be eventually validated.

In the second phase of our investigations, we designed our first sterol, which lacks the methyl groups C19 and C18: 18-19-di-nor-cholesterol, which we called Dchol (see Figure 1) (Róg et al., 2007). To our surprise, this sterol does not induce more order in saturated bilayers than cholesterol does, even though packing of lipid tails’ atoms is almost identical at both sides of Dchol and even slightly higher than in the case of cholesterol. On the contrary, Dchol’s ordering capability is clearly worse. In unsaturated bilayers, the differences were smaller; however, cholesterol was still superior to our artificial Dchol. The molecular level mechanism behind the weaker ordering and condensing effects was related to the larger tilt of Dchol in the bilayer (Aittoniemi et al., 2006). Studies of several sterols have shown that the sterol’s tilt correlates with its ordering capability (Aittoniemi et al., 2006; Khelashvili and Harries, 2013). Thus, our conclusion was that the methyl groups at the β-side are needed to ensure the proper orientation of cholesterol. Following the initial idea, we then designed alternative sterols with the methyl groups removed one by one – we expected that maybe not all of the methyl groups are needed for maintaining the optimal tilt (Pöyry et al., 2008). Contrary to expectations, however, all the designed sterols turned out again to be inferior in their ordering capabilities to cholesterol, although in some cases, the differences were very small. These studies also showed the C18 methyl group to be the most important one, as its removal had the largest effect. Still, other methyl groups also enhanced the sterols’ ordering abilities. All this was very surprising and was in contrast to our expectations, so we continued our investigations even further.

The observation of the most important methyl group being C18 has interesting connotations. The most common lipid chain is an 18-carbon, monounsaturated chain, with the double bond located at position 9–10, and attached at the *sn*-2 position of a glycerol moiety. Cholesterol’s effect on unsaturated lipids is weaker than on saturated ones. However, as our studies have shown (Martinez-Seara et al., 2008), the position of the double bond is significant. The largest differences between saturated and unsaturated lipids were observed when the double bond was located at position 9–10. Shifting the double bond up or down leads to stronger effects of cholesterol, and gradually the interactions of the unsaturated and saturated tails with cholesterol converged. Even shifting an unsaturated tail from the *sn*-2 position to *sn*-1 slightly increased cholesterol’s effects (Martinez-Seara et al., 2009). Plausibly, the reason for this may be the difference in equivalent atom positions in the two tails. Consequently, we proposed an additional function for the C18 methyl group: discrimination between saturated and unsaturated chain. We also hypothesized that lipids and sterols coevolved, leading to the known cholesterol structure, and selection of hydrocarbon chain, which together optimize the desired membrane properties. Moreover, the differences in cholesterol effects on saturated and unsaturated lipids affect phase separation and properties of the formed domains.

**Figure 2:**
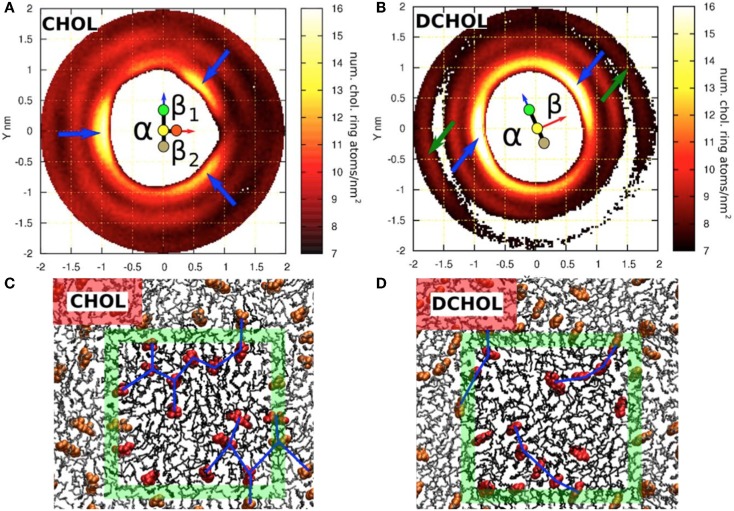
**Sterol–sterol in-plane distribution and configurations of sterol molecules in a DSPC bilayer with 20 mol% sterol**. Two-dimensional density distribution for the ring atoms of **(A)** cholesterol around a tagged cholesterol and **(B)** Dchol around a tagged Dchol. Both **(A,B)** show a schematic representation of the tagged sterol (see also Figure 1). The β-face of cholesterol is divided into two sub-faces: β_1_ and β_2_. **(A)** shows that cholesterols avoid the first coordination shell, instead forming a clear second coordination shell. The three emerging peaks, each on a different face, are marked with blue arrows. **(B)** shows that the two sides of Dchol behave in a similar manner as the smooth α-face of cholesterol. No Dchol is seen in the first coordination shell, and peaks (marked with blue arrows) are observed on both faces. Some structure is still visible in the outer coordination shell around 1.8 nm. Two peaks, which are collinear with the previous ones, are marked with green arrows. This reflects a strong preference to form linear Dchol–Dchol structures. **(C,D)** show a top view of an equilibrated configuration of **(C)** a DSPC/cholesterol bilayer and **(D)** a DSPC/Dchol bilayer. Only one leaflet is drawn for clarity. PC molecules are shown as black sticks and sterols with a red space-filling model. The boundary of the simulation box is marked with the green square and color brightness. **(C)** shows the connections between neighboring cholesterol molecules forming triangular patterns, whereas in **(D)**, the connection patterns formed by Dchol molecules are clearly linear. This fundamental difference is due to the missing out-of-plane methyl groups in the Dchol molecule. Figure adapted from Martinez-Seara et al. (2010).

Another difference between cholesterol and Dchol can be easily visualized. If we look at the cholesterol molecule perpendicularly from its side (Figure 1), we see a clear pattern – a flat and a rough face. Now, if we instead look at the cholesterol molecule from top down, we see a kind of threefold symmetry, shown in Figures 1 and 2. This is caused by the β-face being subdivided into two further faces (Martinez-Seara et al., 2010). Dchol, due to its lack of methyl groups on the β-face, does not display this kind of threefold symmetry. The difference can be visualized well by looking at the two-dimensional radial distribution of cholesterols around a tagged cholesterol shown in Figure 2. This difference may affect the phase behavior of lipid bilayers. As we mentioned above, lanosterol does not promote the *Lo* phase formation, and due to the additional methyl group does not possess the threefold symmetry. As depicted in Figure 2, our preliminary data suggest that the symmetry of cholesterol’s ring affects the sterol–sterol arrangement. Sterols tend to locate in the second coordination shell of each other, with a lipid molecule in between (Martinez-Seara et al., 2010). Due to the threefold symmetry, cholesterol molecules are able to form a fork net (Figure 2) that is likely capable of covering large areas. By contrast, Dchol has only twofold symmetry and thus forms linear structures. It seems plausible that this different form of molecular packing will affect also the phase behavior of Dchol. At this point, we need more extensive studies to further clarify the matter.

## Experiments Confirmed the Results from Atomistic MD Simulations

To validate the results from these MD simulation studies, one first has to synthesize the de-methylated form of cholesterol. This task is not to be taken lightly, as cholesterol has seven chiral centers, which make its synthesis particularly complicated. Nevertheless, Dchol was recently synthesized, 7 years after our first simulation studies of de-methylated sterols (Mydock-McGrane et al., 2014). Synthesis was started from a compound whose synthesis was known before: perhydrochrysenone from which 18-19-di-nor-cholesterol was obtained in eighteen steps. The yield from the whole synthesis was 3.5%, which taking into account the complexity of the process is a very good result.

The properties of Dchol were carefully examined via an extensive set of biophysical methods (Krause et al., 2014). Langmuir monolayers and fluorescence anisotropy measurements showed that Dchol has slightly weaker condensing and ordering ability than cholesterol, in agreement with our simulation data. Calorimetric study showed that the temperature of the main phase transition is within error range for lipid bilayers with both sterol types. Nevertheless, excess heat capacity endotherms showed that cholesterol affects the phase transition more strongly than Dchol, indicating differences in interactions of both sterols with phospholipids. Most interestingly, the results of this study showed decreased nearest neighbor interactions in bilayers with Dchol, compared to those with cholesterol. This result cannot be directly compared to results from MD simulations; however, it has interesting consequences. The difference of nearest neighbor interactions of tens of calories per mole, as observed in this experimental study, might lead to substantial changes in domain size distribution as documented by Monte Carlo simulations (Almeida, 2009).

## Conclusion

Both atomistic MD simulations and experimental studies have shown that cholesterol’s methyl groups are important structural elements of cholesterol and definitely are not molecular fossils. On the contrary, they are important structural elements. Removal of these groups clearly decreases the sterol’s ordering and condensing effects. MD simulation studies have indicated that the decreased ordering is related to a larger tilt of the de-methylated sterols, suggesting that the methyl groups are involved in maintaining the proper orientation of cholesterol in lipid bilayers. Both experimental and MD studies imply that the presence of methyl groups might affect the sterol’s ability to induce phase separation by affecting domain sizes or changing the structure of the formed sterol–lipid–sterol patches. This problem clearly requires more studies, as it might potentially be the most important reason for nature to select cholesterol.

This perspective article has provided a clear example of how MD simulations can independently provide powerful predictions and thus guide experiments. We have shown how constructing molecules that do not exist in nature can increase our understanding of molecular design of lipids and how simulations of these systems are capable of providing correct, valuable predictions later confirmed by experiments. Despite these kinds of successes, the current editorial practice in high-impact journals clearly favors papers that include both MD simulations and experiments. This happens at the expense of pure simulation papers, which are considerably harder to publish. Surely, every model has to be validated with experimental data, yet lifting the requirement that every single simulation study has to be coupled to experiments *in the same paper* might result in the publication of a greater number of progressive, high-quality simulation articles, which would likely provide fresh, valuable ideas, and predictions for experimental scientists.

## Conflict of Interest Statement

The authors declare that the research was conducted in the absence of any commercial or financial relationships that could be construed as a potential conflict of interest.
